# Clinical features and outcomes of bowel perforation in primary pediatric gastrointestinal lymphoma

**DOI:** 10.1186/s12887-021-02944-1

**Published:** 2021-12-04

**Authors:** Jiayu Yan, Yanlong Duan, Tingting Liu, Jianlin Guo, Chunhui Peng, Wenbo Pang, Dan Zhang, Yun Peng, Yajun Chen

**Affiliations:** 1 Department of General Surgery, Beijing Children’s Hospital, Capital Medical University, National Center for Children’s Health, No. 56 Nalishi Road, Xicheng District, CN 100045 Beijing, People’s Republic of China; 2 Medical Oncology Department, Pediatric Oncology Center, Beijing Children’s Hospital, Capital Medical University, National Center for Children’s Health, Beijing, China; 3 Beijing Key Laboratory of Pediatric Hematology Oncology, Key Laboratory of Major Diseases in Children, Ministry of Education, Beijing, China; 4 Department of Emergency Surgery, Beijing Children’s Hospital, Capital Medical University, National Center for Children’s Health, Beijing, China; 5 Department of Radiology, Beijing Children’s Hospital, Capital Medical University, National Center for Children’s Health, Beijing, China

**Keywords:** Primary gastrointestinal lymphoma, Children, Bowel perforation, Surgery, Outcome

## Abstract

**Background:**

Whether surgery can improve the prognosis of patients with primary pediatric gastrointestinal lymphoma (PPGL) who experienced bowel perforation remains controversial. This study aimed to evaluate the prognosis of such patients.

**Methods:**

Nine patients pathologically diagnosed with PPGL who experienced perforation at our center between January 2010 and December 2020 were enrolled and divided into two groups: those with perforation during (*n* = 4) and before (*n* = 5) chemotherapy. Their medical records were reviewed, and long-term follow-up was conducted by telephone in February 2021.

**Results:**

All patients with perforation during chemotherapy were diagnosed with PPGL in the outpatient department. The mean time from outpatient visit to chemotherapy was 17.3 ± 6.1 days. Two patients experienced perforation during the first chemotherapy regimen and received conservative treatment, while the others developed perforation after multiple chemotherapy regimens and underwent surgery. All of the patients received regular chemotherapy and survived for a mean follow-up time of 3.8 ± 1.9 years. No patient with perforation before chemotherapy had a definite diagnosis in the outpatient department. Among these patients, 4 experienced perforation and underwent surgery, of whom 3 developed perforation-related complications and died; the other recurred after chemotherapy. Only the patient who received conservative treatment was diagnosed with PPGL before chemotherapy, received regular chemotherapy, and survived without a recurrence for 1.0 year.

**Conclusion:**

Prompt diagnosis and chemotherapy improve the prognosis of PPGL. Surgery does not affect the prognosis of patients with perforation during chemotherapy but may accelerate disease progression in patients with perforation before chemotherapy.

## Background

Primary malignancies of the gastrointestinal (GI) tract are the most common tumors and are responsible for more than 20% of cancer deaths worldwide but represent less than 5% of all pediatric neoplasms [1, 2]. In contrast to the pattern in adulthood, in which colorectal and gastric cancer rank first and second, lymphoma is the most common GI tract malignancy in childhood, with frequent intestinal involvement [3, 4]. Primary gastrointestinal lymphoma (PGL) mainly involves GI lesions with or without expansion to regional lymph nodes, the spleen, or the liver, according to most studies [2, 3, 5].

The main type of primary pediatric gastrointestinal lymphoma (PPGL) is non-Hodgkin lymphoma (NHL), and its primary treatment is chemotherapy combined with surgery, radiotherapy, immunotherapy or antibiotics when necessary [2, 6]. Bowel perforation is a clinical presentation of patients with PGL. It is also a life-threatening complication during chemotherapy that occurs in 9% of patients with PGL [7]. There have been some reports on the prevention, clinical features, and histopathological features of PGL with bowel perforation in adults [7–10]. Due to the rarity of PPGL, few studies have been conducted on bowel perforation in PPGL. Moreover, there have been no reports on the prognosis after surgery for PGL with perforation, although surgery is generally considered the primary treatment for bowel perforation. Therefore, this study reported the experience of diagnosis and treatment of bowel perforation before and during chemotherapy for PPGL at a single center and evaluated the effect of surgery on prognosis.

## Methods

This study adhered to the ethical principles of the Declaration of Helsinki. The study was approved by the Ethics Committee of Beijing Children’s Hospital (2021-E-054-R). The requirement for informed consent was waived because this study was a retrospective observational study.

We searched the electronic medical records of Beijing Children’s Hospital, Children’s National Medical Center, China, for patients diagnosed with “lymphoma” and “perforation” between January 2010 and December 2020. All clinical data, surgical records and pathologic reports were reviewed by two pediatric surgeons to identify patients who sustained bowel perforation and had pathologically proven lymphoma with involvement of the GI tract. Patients with only radiologic evidence suggestive of gut involvement were excluded.

**Fig. 1:**
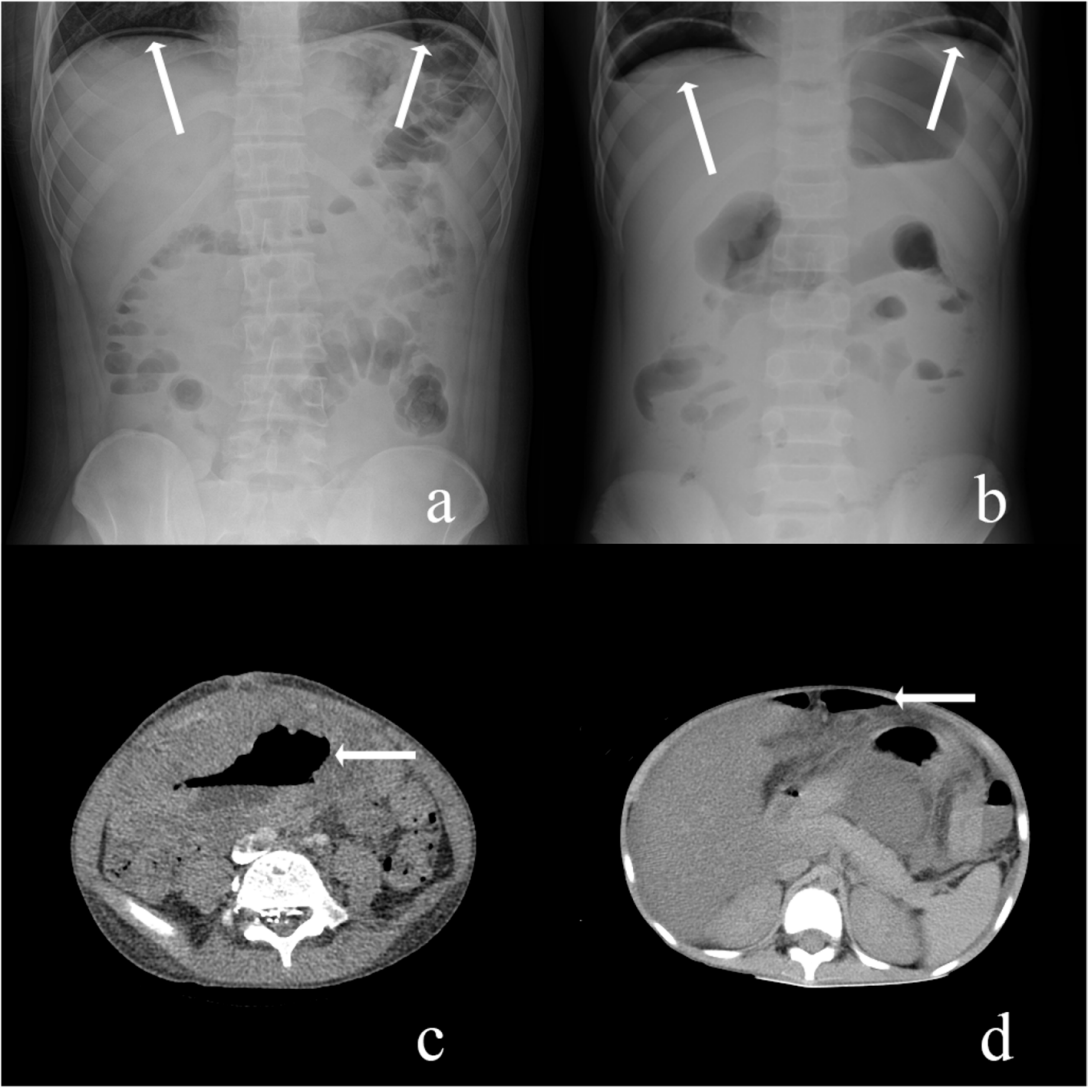
Perforation confirmed by radiology imaging (white arrow: free gas). **a** a little free gas with decreasing under the diaphragm by abdominal X-ray. **b** a lot of free gas without decreasing under the diaphragm by abdominal X-ray. **c** localized intra-abdominal free gas around the colon by abdominal CT. **d** diffuse intra-abdominal free gas in front of the liver by abdominal CT

We stratified the patients into two groups: those who developed bowel perforation before chemotherapy and those who developed bowel perforation during chemotherapy. We collected patient information, including the age of lymphoma diagnosis, site of lymphoma, histopathology, lymphoma classification and stage, date of perforation, site of perforation, surgical information, chemotherapy regimens, date of last chemotherapy treatment before perforation, date and cause of death, and date of last follow-up. Then, we drew the clinical diagnosis and treatment timelines of the two groups. The patients were classified according to the St Jude staging system, which is based primarily on the clinicopathologic features of childhood Burkitt lymphoma and lymphoblastic lymphoma [11, 12]. Bowel perforation was confirmed either intraoperatively or radiologically (defined as the presence of free gas intraabdominally in radiology imaging, Fig. 1) [9]. The criteria for choosing conservative management for patients with bowel perforation included: 1, good general health; 2, no signs of diffuse peritonitis; and 3, localized intra-abdominal free gas according to imaging examination, and no obstruction [13–15]. Close monitoring within the first 24 h of treatment was mandatory to allow early detection of conservative treatment failure. The chemotherapy regimens of all the patients were reviewed and confirmed by a chief hematologist (Table 1). Infection was defined by either a positive culture (blood, tissue, or fluid) for microorganisms or radiologic evidence of infection (consolidation/collection/abscess) [9].

**Table 1:** Chemotherapy regimens

Chemotherapy regimen	Chemotherapy drugs
COP	Cyclophosphamide, Vincristine, Prednisone
COPADM	Cyclophosphamide, Vincristine, Prednisone, Adriamycin, Methotrexate
R + CYVE	Rituximab, Cytarabine, Etoposide
HLH-2004	Cyclosporin A, Dexamethasone, Etoposide

Continuous variables are presented as the mean and SD (normally distributed variables) or median and interquartile range (nonnormally distributed variable). Categorical variables are expressed as numbers and percentages.

## Results

### Patient and tumor characteristics

During the study period, 9 patients met the inclusion criteria, and their clinical characteristics are summarized in Table 2. The mean age at presentation was 9.2 ± 5.4 years, and 7 patients (7/9, 77.8%) were boys. The most common primary site of lymphoma involvement was the small bowel (6/9, 66.7%). Of these patients with perforation, 4 (4/9, 44.4%) were diagnosed with bowel perforation during chemotherapy for lymphoma and 5 (5/9, 55.6%) before chemotherapy. The most common presentation of perforation was abdominal pain (8/9, 88.9%), followed by fever (5/9, 55.6%) and abdominal distension (4/9, 44.4%). When perforation was suspected, abdominal ultrasound (US) was performed in all the patients, with a positive rate of 100.0% (9/9); abdominal X-ray was performed in 6 patients, with a positive rate of 83.3% (5/6); and abdominal computed tomography (CT) was performed in 3 patients, with a positive rate of 66.7% (2/3). The bowel perforations were located at the site of lymphoma involvement in all patients except one who developed a duodenal perforation (with no evidence of viable lymphoma) during treatment with chemotherapy for hemophagocytic lymphohistiocytosis (HLH). Burkitt lymphoma (5/9, 55.5%) was the most common lymphoma associated with perforation, followed by NK/T-cell lymphoma (3/9, 33.3%). According to the St Jude staging system, none of the patients had initial central nervous system and/or bone marrow involvement, and 8 patients (8/9, 88.9%) were diagnosed with aggressive stage III lymphoma.

**Table 2:** Clinical data, histopathology and staging

Case	Age (years)	Sex	Site of lymphoma	Time of perforation	Presentation	Imaging examinations	Site of perforation	Histopathology	Stage^a^
1	6	M	Colon, mesenteric, omentum majus	Diagnosed, during chemotherapy	Ascites	Ultrasound +, CT +	Colon	Burkitt	III
2	3.8	M	Ileocecal, kidney	Diagnosed, during chemotherapy	Abdominal pain, fever, abdominal distension, ascites	Ultrasound +, CT -	Appendix	Burkitt	III
3	5.3	M	Ileum, lesser omentum, peritoneum	Diagnosed, during chemotherapy	Abdominal pain	Ultrasound +, X-ray +	Ileum	Burkitt	III
4	13.7	M	Jejunum, mesenteric, bladder	Diagnosed, during chemotherapy	Abdominal pain	Ultrasound -, X-ray -	Jejunum	Burkitt	III
5	13	M	Jejunum	Undiagnosed, before chemotherapy	Abdominal pain, fever, abdominal distension	Ultrasound +, X-ray +	Duodenum/ Jejunum	NK/T-cell with HLH	II
6	0.9	M	Ileocecal, omentum majus, peritoneum, ligamentum teres hepatis	Diagnosed, before chemotherapy	Fever, abdominal distension, ascites	Ultrasound +, X-ray +	Ileocecal	Burkitt	III
7	9.7	M	Multiple small intestines, regional lymph node	Undiagnosed, before chemotherapy	Abdominal pain, fever, abdominal distension, ascites	Ultrasound +, X-ray +, CT +	Jejunum, ileum	NK/T-cell	III
8	13.5	F	Proximal ileum, mesenteric, omentum majus, ilium	Undiagnosed, before chemotherapy	Abdominal pain, fever	Ultrasound +	Ileum	NK/T-cell with HLH	III
9	16.5	F	Terminal ileum, stomach, rectum, bladder, liver, kidneys, testicle	Undiagnosed, before chemotherapy	Abdominal pain	Ultrasound +, X-ray +	Ileum/Ileum	Diffuse large B-cell	III

^a^ The St. Jude staging classification (Murphy, 1980) for pediatric non-Hodgkin lymphoma

### Patients with bowel perforation during chemotherapy (*n* = 4)

**Fig. 2:**
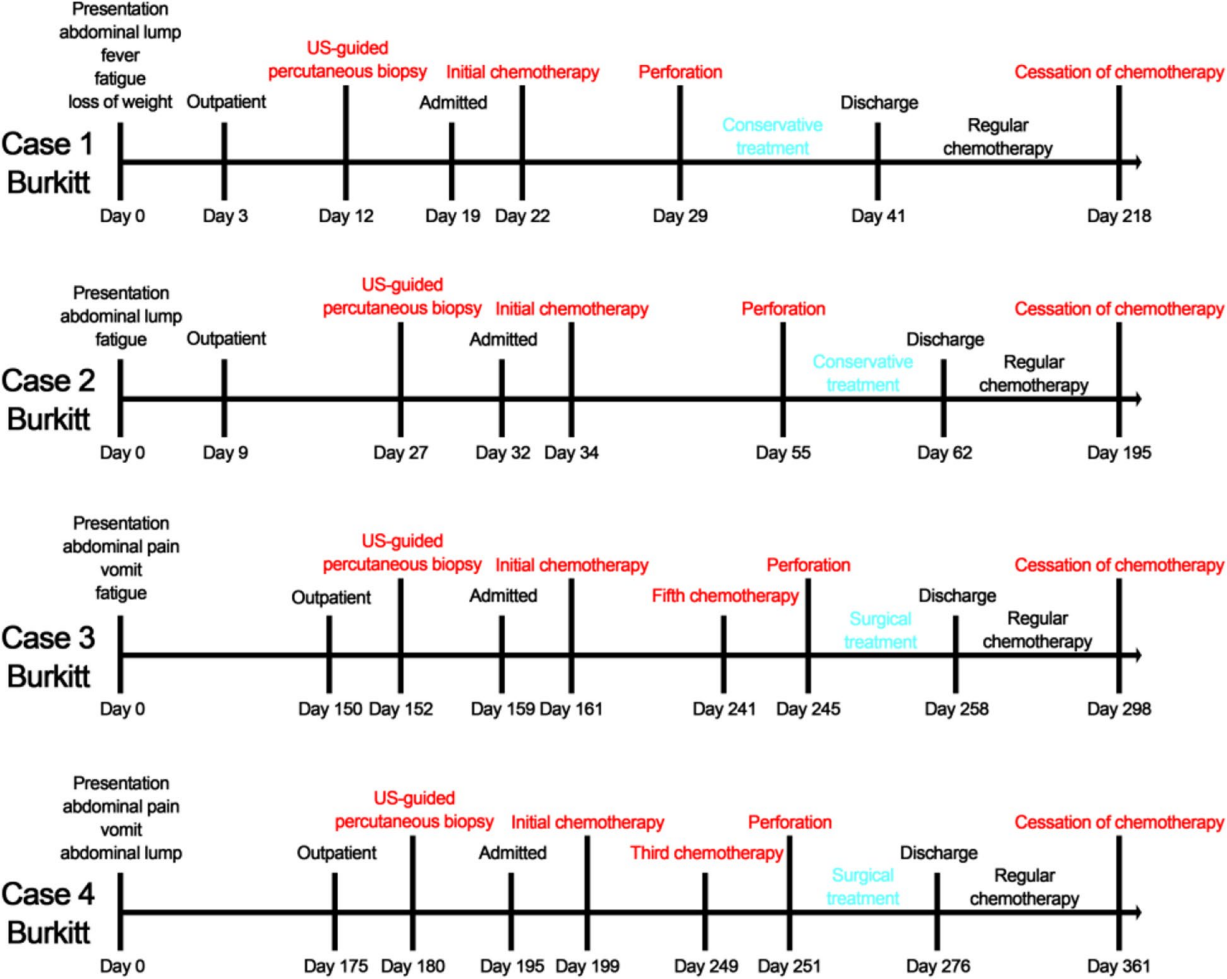
Clinical diagnosis and treatment timelines of PPGL patients who experienced perforation during chemotherapy

All the patients who experienced perforation during chemotherapy underwent US-guided percutaneous biopsy and were diagnosed with Burkitt lymphoma in the outpatient department (Fig. 2). These patients had symptoms for durations of 3, 9, 150, and 175 days. The mean time from the outpatient visit to chemotherapy was 17.3 ± 6.1 days.

In the 2 patients (Cases 1 and 2) who experienced perforation during the first chemotherapy regimen, the duration between the initiation of chemotherapy and perforation was 7 and 21 days, respectively. In contrast, in the patients (Cases 3 and 4) who experienced perforation after multiple chemotherapy regimens, the durations were 4 and 2 days. Case 1 was suspected to have a perforation in the colon based on regular abdominal US evaluation, and abdominal CT confirmed localized intra-abdominal free gas around the colon. Case 2 was suspected to have a perforation in the appendix based on abdominal US performed due to abdominal pain and abdominal distension, but the severity of the presentations did not increase, and abdominal CT during close monitoring revealed no intra-abdominal free gas. Both patients received conservative treatment and subsequent regular chemotherapy, which ended 218 and 195 days after the onset of symptoms. Case 3 was suspected to have an ileal perforation based on abdominal US, confirmed to have free gas under the diaphragm by abdominal X-ray, and ultimately underwent perforated segment resection. Although abdominal US and X-ray did not indicate perforation, following the gradual aggravation of abdominal pain, Case 4 was confirmed to have a jejunal perforation during laparotomy and ultimately underwent perforated segment resection. Cases 3 and 4 then received subsequent regular chemotherapy, which ended 298 and 361 days after the onset of symptoms (Tables 2 and 3).

**Table 3:** Outcomes and follow up

Case	Treatments of perforation	Complications during the treatment of perforation	Follow up time (years)	Status
1	COP, COPADM, perforation, conservative treatment	Pneumonia, PICC-associated bloodstream infection	6.3	Alive
2	COP, COPADM, perforation, conservative treatment	Pneumonia, abdominal infection	2.3	Alive
3	COP, COPADM, R + CYVE, R + CYVE, R + COPADM, perforation, surgical treatment (resection and anastomosis)	Wound infection	4.1	Alive
4	COP, COPADM, R + CYVE, perforation, surgical treatment (resection and anastomosis)	Wound infection	2.3	Alive
5	HLH-2004, perforation, surgical treatment (repair), HLH-2004, surgical treatment (resection and anastomosis)	Pneumonia, wound infection, DIC	–	Died
6	Surgical treatment (open biopsy), perforation	Pneumonia, ATLS, abdominal infection	–	Died
7	Conservative treatment, perforation, surgical treatment (resection, anastomosis and intestinal exteriorization)	EBV infection, abdominal infection, septic shock	–	Died
8	Perforation, conservative treatment, perforation, surgical treatment (resection and anastomosis), HLH-2004	Abdominal infection	2.0	Surgical treatment Chemotherapy Radiotherapy Alive
9	Perforation, conservative treatment, COP, COPADM, perforation, surgical treatment (ileostomy)	Abdominal infection, wound infection	1.0	Alive

### Patients with bowel perforation before chemotherapy (*n* = 5)

**Fig. 3:**
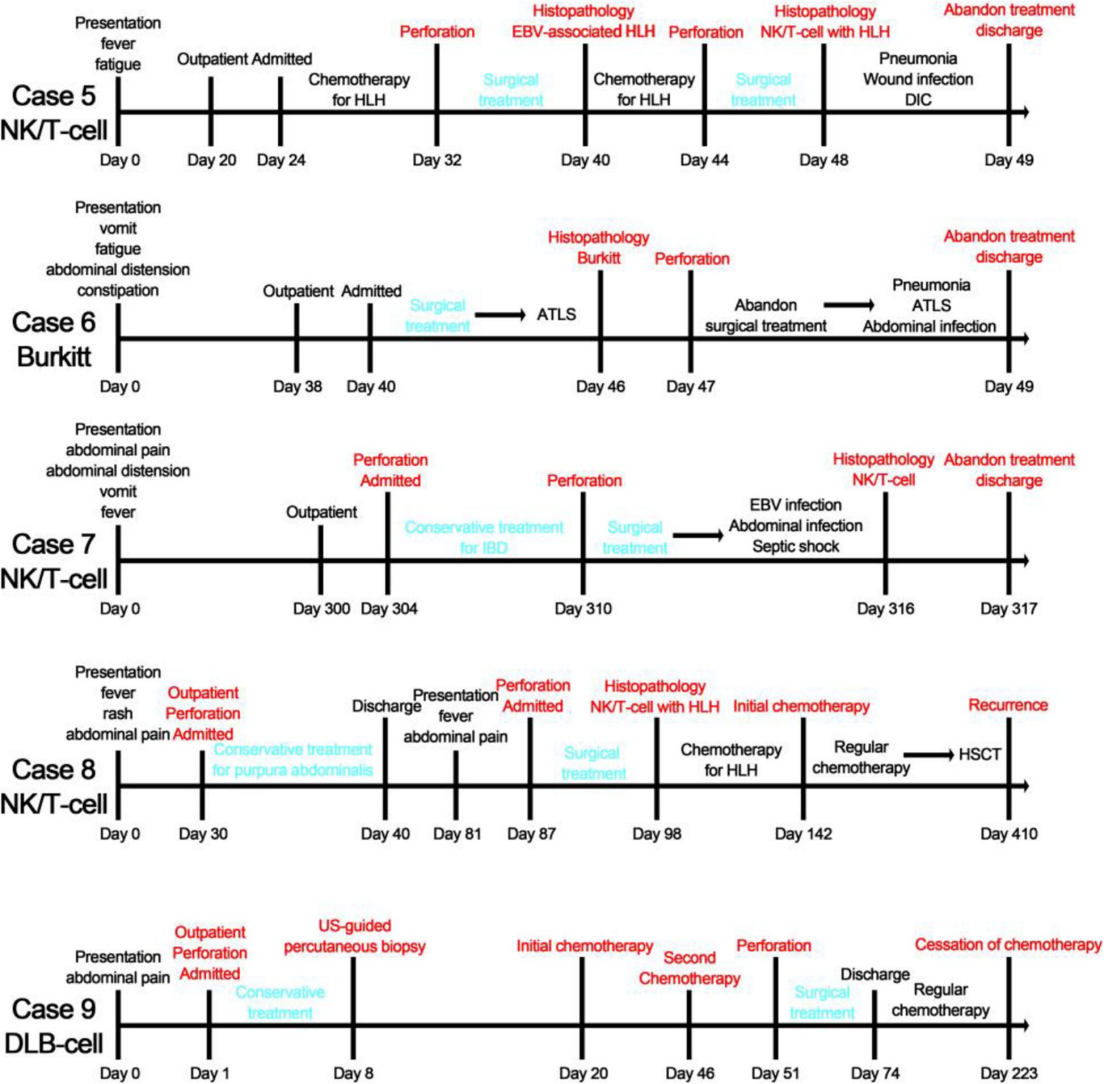
Clinical diagnosis and treatment timelines of PPGL patients who developed perforation before chemotherapy

None of the patients who developed perforation before chemotherapy received had a definite diagnosis before perforation, and 3 (3/5, 60.0%) were misdiagnosed with HLH, inflammatory bowel disease (IBD), and purpura abdominalis in the outpatient department (Fig. 3). The durations of symptoms were 20, 36, 304, 30 and 1 day.

Case 5 was suspected to have two successive perforations after admission based on abdominal US and X-ray during the first chemotherapy regimen for HLH, including a duodenal perforation first and then a new jejunum perforation, which were both confirmed during laparotomy. The patient ultimately underwent duodenal repair and perforated segment resection, respectively. The duration between receiving chemotherapy and the two perforations was 8 and 20 days. Epstein Barr virus (EBV)-associated HLH was diagnosed by postoperative pathology after the first surgery and NK/T cell lymphoma with HLH was diagnosed by postoperative pathology after the second surgery. However, the patient developed pneumonia, wound infection, and disseminated intravascular coagulation (DIC), and was abandoned eventually. Case 6 was diagnosed with intestinal obstruction and underwent an emergency open biopsy. However, the patient developed acute tumor lysis syndrome (ALTS) postoperatively and was suspected to have ileocecal perforation based on abdominal X-ray after being diagnosed with Burkitt lymphoma. The patient developed pneumonia and abdominal infection, and was abandoned eventually. Cases 7, 8 and 9 were admitted with suspected perforation. Case 7 was diagnosed with IBD and confirmed to have jejunum and ileal perforations during laparotomy after receiving conservative treatment for 6 days. However, the patient developed an EBV infection, an abdominal infection, and septic shock postoperatively, and was abandoned eventually. Case 8 was discharged after receiving conservative treatment for purpura abdominalis for 10 days but was suspected to have a new ileal perforation based on abdominal US. The patient ultimately underwent perforated segment resection and was diagnosed with NK/T cell lymphoma with HLH. However, the patient developed nasal lymphoma 410 days after the onset of symptoms despite regular chemotherapy and hematopoietic stem cell transplantation (HSCT). Case 9 underwent US-guided percutaneous biopsy during conservative treatment for perforation after admission. The patient was suspected to have an ileal perforation based on abdominal US, was confirmed to have free gas under the diaphragm by abdominal X-ray during the second chemotherapy regimen for diffuse large B-cell lymphoma, and ultimately underwent ileostomy. The duration between receiving the second chemotherapy treatment and perforation was 5 days. Then, the patient received subsequent regular chemotherapy, which ended 223 days after the onset of symptoms (Tables 2 and 3).

### Outcomes

The treatments for PPGL perforation, the associated complications and overall survival are presented in Table 3. All patients had perforation-related, chemotherapy-related or surgical complications. The most common complication was abdominal infection (5/9, 55.6%), followed by wound infection (4/9, 44.4%). At the time of the last follow-up (February 20, 2021), none of the patients who developed perforation during chemotherapy had died, and the mean survival duration was 3.8 ± 1.9 years from the last chemotherapy treatment. However, among the patients with perforation before chemotherapy, 3 patients who abandoned treatment died within a week of hospital discharge. These patients all died directly due to perforation or subsequent complications. The patient who developed nasal lymphoma underwent further surgical treatment, chemotherapy and radiotherapy and survived for 2.0 years after the last radiotherapy regimen. The last patient was doing well 1.0 years after the last chemotherapy regimen.

## Discussion

As opposed to PGL affecting the stomach in adults, PPGL tends to occur in the small and large intestines, especially in the ileocecal region, making complete resection of tumor tissues possible [7, 16]. The use of surgery for PGL in adults has dramatically decreased in the past few decades, but surgical resection remains the mainstay of treatment for PPGL [17]. Although many small case series have shown improved survival outcomes in children who undergo surgical resection, the largest retrospective study on PPGL reported by Kassira et al. found that surgery had no benefit in children with PPGL younger than 10 years and had adverse effects on survival in patients 10 years or older [2, 18, 19].

Bowel perforation is considered one of the surgical abdomens and should be treated by urgent surgery [20, 21]. The incidence of bowel perforation in PGL is less than 10%, but once it occurs, the patient is more prone to severe complications, such as severe abdominal infection and regional metastasis, which may delay systemic chemotherapy and lead to a worse prognosis [16, 22, 23]. For both physicians and surgeons, the diagnosis and treatment of perforation in PGL remains very challenging. Studies have analyzed the characteristics and prognosis of adult PGL with perforation, but few studies on PPGL with perforation have been reported [7, 10]. In addition, it remains unknown whether surgery improves the prognosis of PGL with perforation.

In this series of consecutive patients with PPGL treated at our center during a contemporary 10-year time period, 9 patients with PPGL experienced bowel perforation. Of them, 4 developed perforations after chemotherapy and 5 developed perforations before chemotherapy, which is consistent with previous literature that nearly half of perforation events occur during the initial presentation of PGL [7]. The sex difference, tumor locations and histopathological types were similar to those in previously published studies about PPGL [2, 6]. However, in contrast to other studies, our study could not obtain the perforation rate in PPGL because our center’s electronic medical record system did not include PGL as a separate diagnosis [7]. In our study, it was only possible to use the keywords “lymphoma” and “perforation” to search for medical records and enroll the 9 patients who were pathologically diagnosed with PPGL and experienced perforation. To the best of our knowledge, our study, although small in sample size, is one of the largest studies to comprehensively describe the clinical characteristics and long-term outcomes of PPGL with perforation.

The evolving literature on PGL has identified that early diagnosis is key to preventing complications and improving prognosis [24]. For instance, a specific diagnosis of the histopathological type before chemotherapy can enable patients to receive appropriate chemotherapy regimens and improve the possibility for a cure [25]. However, the clinical presentation of PGL can vary, ranging from abdominal pain or intestinal obstruction to an occult abdominal lump, which is usually nonspecific and prevents early detection [2, 14]. Our findings are consistent with such reports. Although all patients with perforation after chemotherapy were diagnosed in the outpatient department, there were still 2 patients who had symptoms for nearly half a year before visiting the outpatient department in our center. According to our study, the patients with perforation before chemotherapy in our center did not receive systematic chemotherapy in time. The main reasons are as follows: first, their symptoms were nonspecific, leading to misdiagnosis and mistreatment (Case 5); second, perforation caused by surgery to resolve the obstruction due to PGL resulted in a delay in chemotherapy (Case 6); and finally, surgery was performed after perforation, but severe surgical complications prevented chemotherapy (Case 7), or chemotherapy was delayed due to surgery (Case 8). Surgery plays a limited role in diffuse cases, such as for patients with perforation before chemotherapy, although it may be required to obtain an accurate diagnosis. These findings emphasize the need to obtain a diagnosis as early as possible before surgery and chemotherapy, either by US- or CT-guided percutaneous biopsy [8].

It appears that, in patients with PPGL, in addition to tumor location being found to be a significant predictor of survival, histopathological variant appears to be a predictor of survival: Burkitt lymphoma and diffuse large B-cell lymphoma have been reported to be associated with better overall survival than other histologies [2, 6]. In the current study, all patients with perforation during chemotherapy had Burkitt lymphoma, and they all received regular chemotherapy and survived, regardless of whether they had undergone surgery for the perforation. However, of the patients with perforation before chemotherapy, 3 had NK/T cell lymphoma, 1 had diffuse large B-cell lymphoma, and the remaining one had Burkitt lymphoma. These observations seem to suggest that patients with Burkitt lymphoma tend to develop perforations during chemotherapy and rarely present with perforation as an initial symptom. In addition, tumor stage was not found to affect patient prognosis in the current study, which is consistent with the findings of Kassira et al. [2].

Perforations often occur within the 4 weeks of the first cycle of chemotherapy [9]. Tumor necrosis and inflammation after the administration of chemotherapy contribute to later perforation during chemotherapy [7]. The present study reveals that if we can identify localized perforations during chemotherapy based on symptoms (such as ascites and fever) and abdominal B-ultrasound or CT findings (localized intraabdominal free gas), we can attempt conservative treatment by adjusting the low-dose chemotherapy regimen to reduce the occurrence of diffuse perforations and the risk of complications arising from surgery for perforation. Moreover, we hope that the objective risk scoring system developed by Aoki et al. can be used to predict the risk of perforation before each cycle of chemotherapy, reduce peritoneal contamination from perforation by elective surgery or facilitate early recognition and avoid perforation by adjusting the chemotherapy regimen, such as rituximab-based chemotherapy regimens, in advance [15, 26, 27].

Similar to our main findings, infection was the most common complication of perforation in patients with PGL, followed by wound infection due to surgery for perforation, which can both interrupt subsequent chemotherapy and affect patient survival [2, 7, 8]. Furthermore, since systemic chemotherapy can attenuate immune responses, more severe complications, including septic shock, DIC, and even multiorgan failure, can occur if the infection is followed by perforation and surgery, as occurred in Cases 5 and 7 in our study, who had poorly controlled complications.^9^ These problems are extremely difficult to address, and the majority of deaths were attributable to these complications, as our research confirms.

Most studies have addressed perforation characteristics, preventive measures and outcomes during chemotherapy, but few studies have focused on perforations before chemotherapy [7, 9, 10]. This may be related to the inability to intervene in advance for these perforations [7]. We also failed to intervene for these perforations before chemotherapy. Thus, we focused on the treatment process after admission. Through the evaluation of clinical diagnosis and treatment timelines, patients with perforation before chemotherapy were compared with those with perforation during chemotherapy in this study. The results emphasized the importance of prompt diagnosis and chemotherapy in improving prognosis and confirmed that surgery did not improve the outcomes of PPGL with perforation before chemotherapy and seemed to accelerate the disease progression. Further studies involving larger sample sizes are required to verify the effect of surgery on perforations before chemotherapy.

This study has several limitations. First, the retrospective analysis of a highly selective group of patients might have resulted in selection bias. Second, we did not include treatment-related variables in the analysis. Some patients underwent adjusted chemotherapy, and others were required to receive total parenteral nutrition and bowel rest during chemotherapy, which might have affected the results. Prospective studies are required to investigate whether patients with localized bowel perforation can be treated conservatively. Third, perforations occurred in only 9 PPGL patients, limiting our ability to draw conclusions regarding the timing of surgery and the need for a simultaneous enterostomy in PPGL patients who undergo perforated segment resection. Multicenter studies are expected in the future.

## Conclusions

Prompt diagnosis and chemotherapy improve the prognosis of PPGL. In patients with perforation during chemotherapy, surgery may not affect patient prognosis. In patients with perforation before chemotherapy, surgery plays a role in confirming the diagnosis but has limited effects on the treatment and may accelerate the progression of the disease.

## Data Availability

All data generated or analyzed during this study are included in this published article.

## Abbreviations

GI: Gastrointestinal
PGL: Primary gastrointestinal lymphoma
PPGL: Primary pediatric gastrointestinal lymphoma
US: Ultrasound
CT: Computed tomography
HLH: Hemophagocytic lymphohistiocytosis
IBD: Inflammatory bowel disease
EBV: Epstein Barr virus
DIC: Disseminated intravascular coagulation
ALTS: Acute tumor lysis syndrome
HSCT: Hematopoietic stem cell transplantation
